# Editorial for the Special Issue on Microfluidics and Lab-on-a-Chip Applications for Biosensing

**DOI:** 10.3390/mi13122060

**Published:** 2022-11-24

**Authors:** Laura Cerqueira, João Mário Miranda

**Affiliations:** 1 LEPABE—Laboratory for Process Engineering, Environment, Biotechnology and Energy, Faculty of Engineering, University of Porto, Rua Dr. Roberto Frias, 4200-465 Porto, Portugal; 2 ALiCE—Associate Laboratory in Chemical Engineering, Faculty of Engineering, University of Porto, Rua Dr. Roberto Frias, 4200-465 Porto, Portugal; 3 CEFT—Transport Phenomena Research Center, Faculty of Engineering of University of Porto, Rua Dr. Roberto Frias, 4200-465 Porto, Portugal

Microfluidics refers the use of interdisciplinary science and engineering concepts that can control and manipulate small fluidic volumes. Its integration into lab-on-chip devices allowed for a resounding evolution in research, with widespread applications in pharmaceutical and life science research and environmental, industrial, and food safety areas. The introduction of miniaturization offers enhanced versatility, ease-of-use, time-to-result, and reduced costs per test, hence benefitting both society and the business sector.

In this context, in this Special Issue, we intended to bring to light the development of novel designs for miniaturization and biosensors, and to show its broader potential usage in different research fields. Three original research papers and two review articles were published, focusing mostly on biosensing technologies [1,2], detection [3,4] and macromolecular delivery systems [5].

In particular, Ren et al. [1] reported a narrow straight microchannel array for the analysis of transiting speed of floating cancer cells. Circulating tumor cells (CTCs) have been considered as an indicator in cancer diagnosis, as a decisive step in the formation of a secondary tumor. The authors created an analytical model that describes cellular transiting speed with deformation along a constricted microchannel. The developed microfluidic model mimics the conditions for cell deformability in the bloodstream and is an effective tool to overcome the challenges associated with observing cells in vivo conditions and low throughput and low imaging quality.

Zhao et al. [2] review the state of the art of whispering-gallery-mode (WGM) microbubble sensors. WGMs are a kind of miniature optical resonators, and out of the different types of WGMs, the authors focus their review on microbubble WGM resonators. In microfluidic applications, the sensor is a transparent microchannel with a thin wall and an enlarged region, which is usually made of silica or glass. Small changes in properties such as temperature, pressure, composition, refractive index or flow rate can be optically detected when the flow passes through the microbubble region, as the intensity and frequency of the light passing through the microbubble region also change. The review discusses the different sensing mechanisms used by microbubble sensors, the different fabrication methods and their main applications. The review concludes by summarizing the practical applications and current technology limitations of WGM microbubble sensors.

Digital polymerase chain reactions (dPCR) can be used to quantify the concentration of a given DNA sequence in a sample. In this technique, the sample is divided into a large number of droplets, with each one operating as a microreactor where the polymerase chain reaction takes place. Chen et al. [3] developed a dPCR platform based on nanoliter droplets produced in a static droplet array. The system has a relatively low sample loading time (10 s). The PCR system was pressurized to avoid sample evaporation. A hepatitis B virus (HBV) plasmid was selected as the target of DNA amplification by the PCR platform. The experimental results show that the platform is able to successfully quantify DNA concentrations.

Raju et al. [4] focused on the state of the art of exosome isolation and detection platforms. Exosomes are extracellular vesicles that are spherical particles enclosed by a phospholipid bilayer. They are present in many biological fluids and play a crucial role in intercellular communication by transporting and delivering cargo between their cells, promoting disease progression. At first, the authors presented an overview of the traditional approaches for exosome isolation based on their physical properties, such as density, size, surface component and precipitation. Subsequently, they explained the advantages of lab-on-a-chip approaches, considering the drawbacks of the traditional methods. They discussed the methods based mostly on immunoaffinity approaches and nanoplasmonics detection. In spite of the interesting features that have contributed to the clear progression of the microfluidic isolation and detection methods compared to the conventional strategies, the authors pointed out that most of these first-generation approaches must continue to evolve in order to play a full role in clinical analysis. The main reasons for this include the lack of standardization and validation of microfluidic methods, the relatively low processing capacity and the complexity of all the biological samples.

Analysis of cell content (e.g., DNA, RNA and proteins) or delivery of cargo to a cell may require the disruption or permeabilization of the cell membrane to release the cell’s contents into a solution for further processing or the delivery of particles or macromolecules to the cell. Thus, methods for cell membrane disruption are in demand. Liu et al. [5] developed a platform for sonoporation of cell membranes that uses ultrasounds combined with a microbubble attached to the cell membrane wall. The paper explores the use of a traveling surface acoustic wave (TSAW) device to induce cell disruption using a non-cavitating microbubble. The authors analyze the effect of input voltage and the number of microbubbles on cell sonoporation. They also discuss the physical mechanism of sonoporation and report that the microbubbles were deformed due to an acoustic radiation force and induced cell membrane deformation, leading to reversible perforation in the cell membrane.
